# Hallmarks of crustacean immune hemocytes at single-cell resolution

**DOI:** 10.3389/fimmu.2023.1121528

**Published:** 2023-01-24

**Authors:** Fan Xin, Xiaobo Zhang

**Affiliations:** ^1^ College of Life Sciences, Zhejiang University, Hangzhou, China; ^2^ Laboratory for Marine Biology and Biotechnology of Pilot National Laboratory for Marine Science and Technology (Qingdao), Qingdao, China; ^3^ Southern Marine Science and Engineering Guangdong Laboratory (Zhuhai), Zhuhai, China

**Keywords:** single-cell RNA sequencing, hemocytes, crustaceans, innate immunity, immune response

## Abstract

In invertebrates, hemocytes are the key factors in innate immunity. However, the types of invertebrate immune hemocytes are unclassified due to the limitation of morphological classification. To determine the immune hemocytes of crustaceans, the heterogeneity of hemocytes of shrimp *Marsupenaeus japonicus* and crayfish *Procambarus clarkii*, two representative crustacean species, were characterized in this study. The results of single-cell RNA sequencing indicated that shrimp and crayfish contained 11 and 12 types of hemocytes, respectively. Each of different types of hemocytes specifically expressed the potential marker genes. Based on the responses of shrimp and crayfish to the infection of white spot syndrome virus (WSSV) and the challenge of lipopolysaccharide (LPS), four types of immune hemocytes of crustaceans were classified, including semi-granular hemocytes involved in antimicrobial peptide production, granular hemocytes responsible for the production of antimicrobial peptides, hemocytes related to cell proliferation and hemocytes in immunity-activated state. Therefore, our study provided the first classification of crustacean hemocytes as well as of immune hemocytes of crustaceans at the single-cell resolution, which would be helpful to understand the innate immunity of invertebrates.

## Introduction

Cells of the hematopoietic system are essential for maintaining the homeostasis of the organism, notably by participating in the immune responses, removing apoptotic cells, and producing various cytokines or clotting factors, especially for invertebrates which rely dramatically on hematopoietic system for innate immunity (1). In vertebrates, the composition and functions of blood cells are well characterized. The red blood cells of vertebrates mainly transport oxygen and carbon dioxide, and buffer the acid-base balance in the body (2), while white blood cells serve as the first-line defenders of inflammatory reactions and play very important roles in immune responses (3). Platelets are involved in the process of blood coagulation, anticoagulation and fibrinolysis (4). For invertebrates, however, there is only a simple morphological classification of hemocytes (5). At present, the knowledge of hematopoietic system for invertebrates is very limited, most of which results from insects. In total, eight types of hemocytes are found in insects, including prohemocytes, plasmatocytes, granular cells, coagulocytes, crystal cells, spherulocytes, oenocytoids and thrombocytoids, which are defined by their morphology with light microscopy or transmission electron microscopy (6). But the majority of insects do not possess all types of hemocytes. In *Drosophila*, there are only lamellocytes, crystal cells and plasmatocytes (7). Plasmatocytes are the most common hemocyte similar to macrophages in vertebrates. They are usually involved in phagocytosis (7). Granular cells have a small nucleus and many granules in the cytoplasm (6). Coagulocytes participate in the clotting process (6). Spherulocytes display a variety of different shapes with numerous small spherical inclusions (8). Oenocytoids are responsible for melanization and phenoloxidase production (8). Recently, single-cell genomics analysis of differentially expressed genes in mosquito identifies the transcriptional signatures of two cell types (9). At present, however, the blood cells of invertebrates have not been well classified.

The immune responses of invertebrates completely depend on innate immunity, which consists of two highly interconnected components, the humoral and the cellular responses (10). The humoral defense is composed of soluble effector molecules such as anti-microbial peptides, complement-like proteins, melanin and the phenoloxidase (PO) pathway, most of which can be manufactured in and secreted by hemocytes (11). The cellular response can be mediated by hemocytes, such as phagocytosis, encapsulation, nodulation and apoptosis (10). Hemocyte-mediated phagocytosis is enabled by opsonization of microorganisms with complement-like proteins and the pathogens can be engulfed and killed by a membrane-bound enzyme system in hemocytes (12). In addition to their roles in innate immunity, hemocytes also play essential roles in tissue modeling and are indispensable for embryonic development in *Drosophila* (13). Although hemocytes play important roles in innate immunity and embryonic development in invertebrate, the pathways underlining their differentiation into the possible classes of cells remain unknown. Whether the current classification represents true cell types or states, and if cell subpopulations also exist in invertebrates, need to be further explored.

Over the past years, the revolution of single cell transcriptomics has enabled an unbiased quantification of gene expressions between individual cells (cell-to-cell variation) (7). It has become feasible to analyze the profiles of single cell transcriptomics and the possible function of each cell (7). To identify the types of hemocytes of invertebrates, single-cell sequencing was used to analyze the hemocytes of crustaceans shrimp and crayfish. Shrimp and crayfish, the species of invertebrates living in pathogens-rich environment, have evolved strong innate immunity, which mainly depend on the hemocytes (blood cells). An open body cavity and abundant content of hemolymph of shrimp and crayfish individuals make them the best candidates for analyzing the cell types and immune functions of invertebrate hemocytes by single-cell RNA sequencing. The results showed that the immune hemocytes of crustaceans could be classified into four types including semi-granular hemocytes involved in antimicrobial peptide production, granular hemocytes responsible for the production of antimicrobial peptides, hemocytes related to cell proliferation and hemocytes in immunity-activated state.

## Materials and methods

### Shrimp and crayfish culture

*Marsupenaeus japonicus* shrimp and *Procambarus clarkii* crayfish about 40-50g each was cultured in groups of 5 individuals in tanks containing aerated seawater and fresh water at room temperature, respectively. To ensure the absence of WSSV in shrimp and crayfish prior to experimental infection, PCR was performed using WSSV-specific primers (5’-TATTGTCTCTCCTGACGTAC-3’ and 5’-CACAT TCTT CACGAGTCTAC-3’). Virus-free shrimp or crayfish were challenged with phosphate-buffered saline (PBS), WSSV (10^5^ copies/ml) or lipopolysaccharide (LPS) (4mg/kg for shrimp and 1mg/kg for crayfish) by injection into the lateral area of the fourth abdominal segment. At different time after challenge, the shrimp and crayfish hemocytes were collected.

### SMRT (single-molecule real-time) library construction, sequencing and sequence analysis

Total RNAs were extracted from shrimp or crayfish hemocytes using TRIzol LS reagent (Invitrogen, USA) following the manufacturer’s instructions. Genomic DNA was removed by DNase I (Invitrogen, USA) digestion. To get the full-length transcriptome of shrimp or crayfish hemocytes, SMRT sequencing was conducted, which allowed direct sequencing of full-length, single-molecule cDNA sequences with a read length of up to 70 kb, avoiding the misassemble of short reads by second generation sequencing. Briefly, the extracted intact RNA (10 μg) was reversely transcribed into cDNA using a SMARTer PCR cDNA synthesis kit (Takara, Japan) following the manufacturer’s protocols. Size fractionation and selection were performed using the BluePippin size selection system (Sage Science, USA). The library was sequenced in three SMRT cells on a PacBio RSII platform using C4 reagents and 3-4 h sequencing movies.

The PacBio SMRT analysis software v2.3.0 (http://www.pacb.com/products-andservices/analytical-software/smrt-analysis/) was used to filter out low-quality reads (read length<50 bp and read score<0.75). The reads generated multiple passes of the inserts. To improve the accuracy of subreads sequences, the consistency of multiple subreads from the same reads was corrected, generating a unique read, known as circular consensus sequencing (CCS) reads. If a read possessed 5’-cDNA primer, 3’-cDNA primer and polyA tail preceding the 3’-cDNA primer, it was considered to be a potential FLNC (full-length non-chimeric) read. After removing the 5’- and 3’-cDNA primers and polyA tail using the Pac-bio recommended procedure (https://github.com/PacificBiosciences/IsoSeq.3), the FLNC reads were obtained. In order to remove the redundancy in FLNC, CD-HIT (clustered database-hit) was first introduced and used to cluster the FLNC to remove redundancy. Subsequently cogent (coding genome reconstruction tool) analysis (https://github.com/Magdoll/Cogent) was conducted using K-mer similarity profiles to partition full-length coding sequences into gene families and the remaining sequences were considered as transcripts expressed in shrimp or crayfish hemocytes.

### Functional annotation of transcripts

Functional annotation matching each unique transcript was performed by searching Nr, Swiss-Prot, COG (clusters of orthologous group) and KEGG (Kyoto encyclopedia of genes and genomes) databases using BlastX with an E-value cut-off of 10^−5^. Protein function was predicted based on the annotation of the most similar hit across all databases. The unique transcripts identified by BlastX were submitted to blast2GO v4.1 (http://www.blast2go.com) to assign GO (gene ontology) categories. To identify the protein coding potential of each unique transcript, the open reading frames (ORFs) within unique transcripts were predicted using TransDecoder v2.0.1 (https://transdecoder.github.io) with default parameters.

### Isolation and viability of hemocytes

The hemocytes of a single shrimp were collected using the modified anticoagulation buffer (450mM NaCl, 10mM KCl, 10mM EDTA (ethylene diamine tetraacetic acid), 10mM HEPES (N-2-hydroxyethylpiperazine-N-ethane- sulphonicacid), pH 7.45) at a ratio of 2 (anticoagulation buffer):1 (hemocytes). The hemocytes of a single crayfish were collected using PBS containing 10 mM EDTA (pH 4.6). All the samples were examined by trypan blue to determine the viability of hemocytes. Only the samples with a hemocyte viability >80% were subjected to single cell sequencing.

### Single cell RNA sequencing

The hemocyte suspension was loaded into Chromium microfluidic chip with V3 chemistry and barcoded with a 10×Chromium Controller (Illumina, USA). RNAs from the barcoded hemocytes were reversely transcribed and then sequencing libraries were constructed with reagents from a Chromium Single Cell V3 reagent kit (10× Genomics) according to the manufacturer’s instruction (Illumina, USA). Sequencing was performed with Illumina NovaSeq 6000 according to the manufacturer’s manual (Illumina, USA) and the data were collected.

FastQC was performed to control the quality of the raw reads. Generally, FASTQ files from raw base call generated by Illumina sequencing were designated as input file. Raw read sequences were pre-processed through Trimmomatic software to remove low-quality reads, trailing low quality and adapters. The remaining reads that passed all the filtering steps were counted as clean reads for further analysis.

### Cell clustering based on gene expression

In order to reduce the gene expression matrix to its most important features, Cell Ranger used principal components analysis (PCA) to change the dimensionality of the dataset from (cells×genes) to (cells×M) where M was a user-selectable number of principal components (via num_principal_comps). For visualizing data in 2-dimentional space, Cell Ranger passed the PCA-reduced data into t-SNE (Stochastic neighbor embedding), a non-linear dimensionality reduction method (14). The runtime was also decreased by fixing the number of output dimensions at compile time to 2 or 3.

After raw reads were demultiplexed by t-SNE, they were mapped to the transcriptome of shrimp or crayfish by 10×Genomics Cell Ranger pipeline (https://support.10×genomics.com/single-cell-geneexpression/software/pipelines/latest/what-is-cell-ranger). Basically, cell ranger reanalysis took feature-barcode matrices produced by cellranger count or cellranger aggr and reruned the dimensionality reduction, clustering, and gene expression algorithms using cellranger default parameter settings. The seurat package was then used for data normalization, dimensionality reduction, clustering, differential expression. Seurat alignment method including canonical correlation analysis (CCA) for integrated analysis of datasets was used. For clustering, highly variable genes were selected and the principal components based on those genes used to build a graph, which was segmented with a resolution of 0.6. In brief, for each gene and each cell barcode (filtered by CellRanger), unique molecule identifiers were counted to construct digital expression matrices. Subsequently, secondary filtration by Seurat was performed as a gene with expression in more than 3 cells was considered as expressed, and each cell was required to have at least 200 expressed genes.

### Data integration

For the integration analysis of the single-cell sequencing data from healthy, WSSV-challenged and LPS-treated shrimp or crayfish, the datasets were combined using the R based toolkit Seurat v3 (15). Briefly, the datasets were normalized. Subsequently, the anchors common between the datasets and the combined objects were used to perform canonical correspondence analysis to correct for the batch effect which was followed by data integration to carry out Z-score normalization. The integrated data were clustered using principal component analysis and visualized with t-distributed Stochastic Neighbor Embedding (t-SNE). To evaluate the difference of hemocyte clusters between treatments, 2-fold change between different clusters was considered to be significant (16, 17).

## Results

### Identification of the clusters of shrimp hemocytes by single-cell RNA sequencing

**Figure 1:**
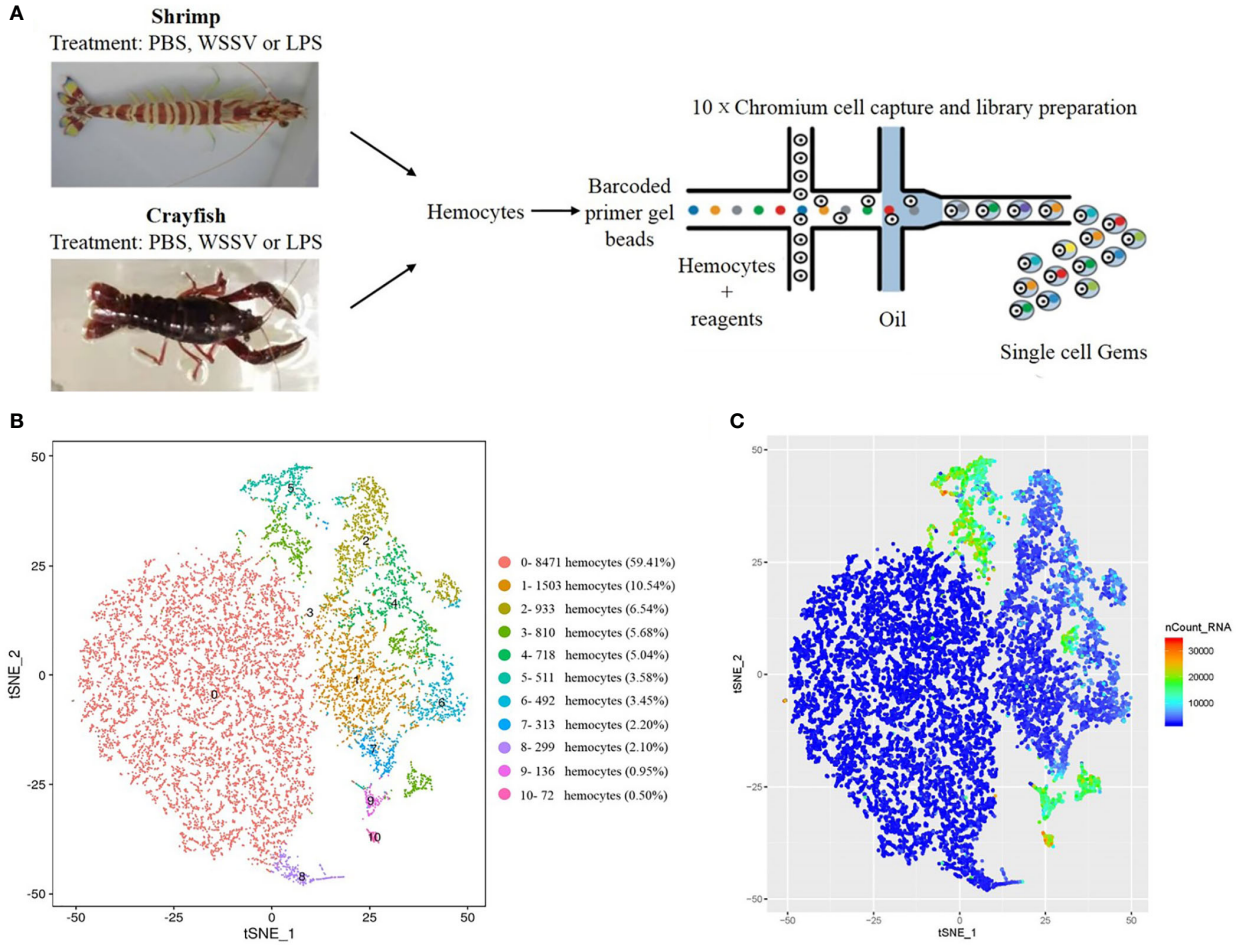
Identification of the clusters of shrimp or crayfish hemocytes by single-cell RNA sequencing. **(A)** Schematic diagram highlighting the experiments for the isolation of shrimp or crayfish hemocytes and the sequencing of single-cell RNAs using the 10×chromium system. **(B)** The clusters of hemocytes by t-SNE analysis. A total of 14,258 hemocytes were characterized. The different types of hemocytes were indicated with different colors. The axes corresponded to the 2-dimensional embedding produced by the t-SNE algorithm. **(C)** t-SNE plot of 14,258 hemocytes colored by UMI counts. The hemocytes with greater UMI counts likely had higher RNA content than the hemocytes with fewer UMI counts. The axes corresponded to the 2-dimensional embedding produced by the t-SNE algorithm. Pairs of hemocytes that were close to each other had more similar gene expression profiles than the hemocytes that were distant from each other.

To identify the types of shrimp hemocytes, single-cell RNA sequencing was conducted (**Figure 1A**). Because of the lack of complete sequence of shrimp genome, the full-length transcriptome of shrimp hemocytes was characterized using single-molecule real-time (SMRT) sequencing prior to single-cell RNA sequencing. A total of 722,493 raw reads (43.06 gigabases) were obtained. After removing adaptor sequences, low-quality sequences and short sequences (<50 bp), 18,680,499 clean reads remained. Totally, 592,794 CCS (circular consensus sequence) reads with an average length of 3,169 bp (**Supplementary Table S1**) were further obtained. Of the 592,794 CCS reads, 408,293 were identified as full-length non-chimeric (FLNC) reads (**Supplementary Table S1**). After removing redundancy, 13,007 genes, including 1,225 lncRNAs, were obtained (GenBank accession number PRJNA894118) (**Supplementary Table S1**).

Although the sequence of shrimp genome (*Litopenaeus vannamei*) was reported (18), more than 50% of the genes from our single-molecule real-time sequencing could not be found in the sequenced shrimp genome database. The analysis indicated that 82% reads of our single-molecule sequencing could match the full-length transcriptome of shrimp hemocytes, indicating that the shrimp transcriptome could cover most of the expressed genes in the hemocytes of *M. japonicus*.

To annotate the gene function, the sequences of shrimp transcriptome were searched against the databases including NR (non-redundant protein database), NT (nucleotide sequences), Pfam, COG (cluster of orthologous groups of proteins), Swiss-prot, KEGG (Kyoto encyclopedia of genes and genomes) and GO (gene ontology). The results showed that of the 13,007 coding genes (including protein-encoding genes and lncRNAs), 11,124 (85.52%, E-value ≤ 10^−5^) genes could be at least annotated in one database (**Supplementary Figure S1A**). Of the top 20 species with matches, 36.21% of the hits were from the *hyalella azteca* and 5.4% from *Marsupenaeus japonicus* (**Supplementary Figure S1B**). The GO analysis indicated that 158,11, 8,775 and 9,509 genes were enriched in biological processes, molecular functions and cellular components, respectively (**Supplementary Figure S1C**). As revealed by KEGG analysis, the shrimp genes played important roles in biological functions including cellular processes, environmental information processing, genetic information processing, human diseases, metabolism and organismal systems (**Supplementary Figure S1D**). The most enriched cellular signaling pathway was signal transduction.

To conduct the single-cell RNA sequencing, 14,258 hemocytes were captured and deeply sequenced using the 10×chromium platform (**Figure 1A**). The t-distributed stochastic neighbor embedding (t-SNE) analysis indicated that the 14,258 hemocytes could be divided into 11 clusters (named from SC0 to SC10), the largest of which contained 8,471 hemocytes (59.41%), while the smallest hemocyte population contained only 72 hemocytes (0.50%) (**Figure 1B**) (GenBank accession no. PRJNA913773). Currently, the types of crustacean hemocytes are not exactly clear although it was suggested that they could be divided into hyaline cells, semi-granular cells and granular cells (GCs) (19). The 11 clusters of hemocytes greatly exceeded the known types of invertebrate hemocytes. The overall gene expression of these clusters was then determined by UMI (unique molecule identifier) counts. The data revealed that clusters 3, 5, 9 and 10 had higher gene expression levels, while the overall expression levels of clusters 0, 1 and 8 were relatively lower (**Figure 1C**), indicating the complexity of gene expressions in the hemocyte clusters.

### Functional analysis of shrimp hemocyte clusters

**Figure 2:**
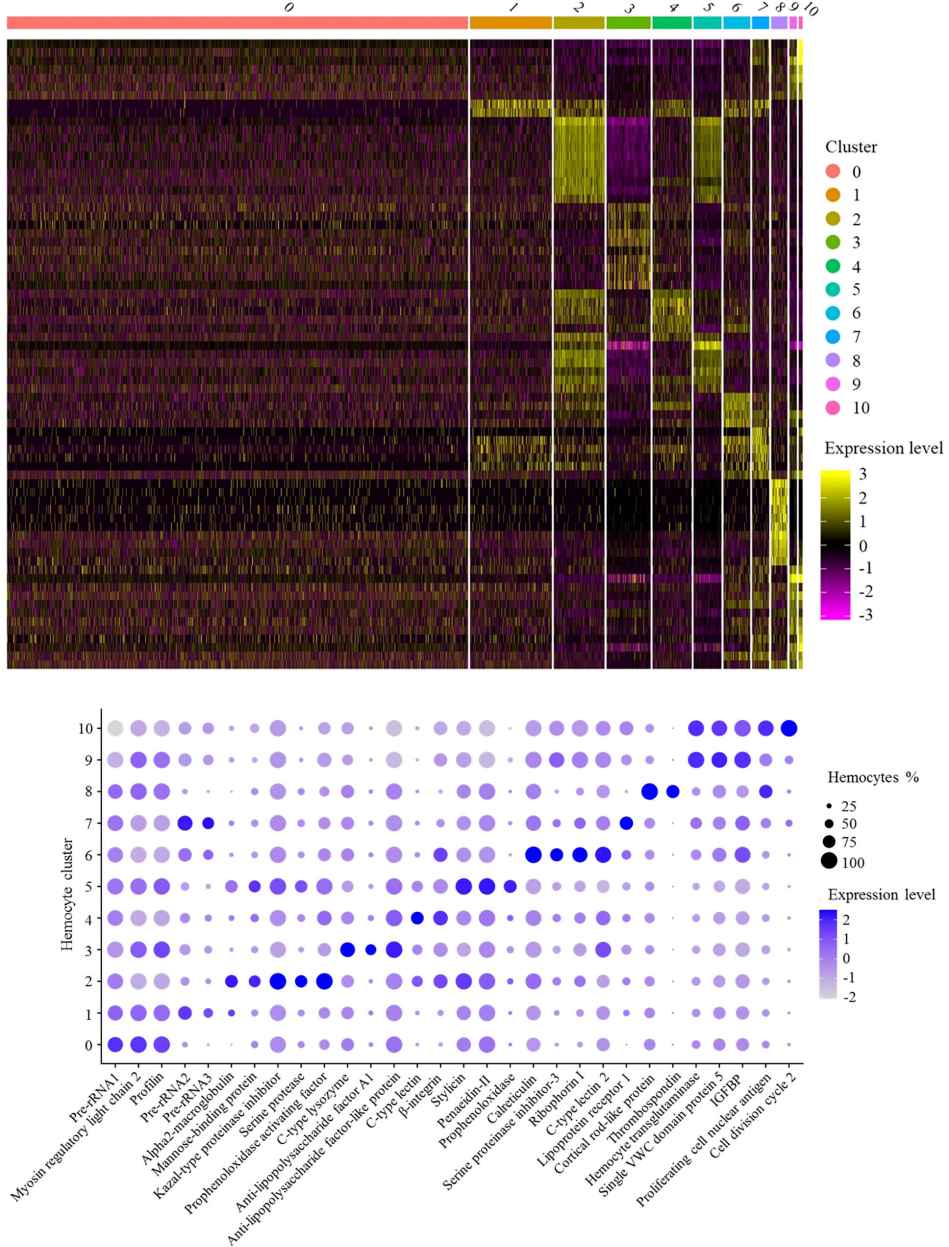
Functional analysis of shrimp hemocyte clusters. **(A)** Heat map of expression levels of the top 10 highly expressed genes in each hemocyte cluster. Each column represented a hemocyte cluster and each row indicated a gene. **(B)** Dot plots of genes highly expressed in 11 hemocyte clusters. Color gradient of the dot represented the expression level, while the dot size indicated the percentage of hemocytes expressing the genes. IGFBP, insulin-like growth factor-binding protein.

Due to the lack of markers of invertebrate hemocytes, differential expression analysis of genes from 11 clusters of shrimp hemocytes was conducted to further characterize the hemocyte types of 11 clusters. Based on filtering criteria of UMI (unique molecular identifier)>1, log2 fold change>0.25 and *p*<0.05, the relative highly expressed genes of each hemocyte cluster were obtained. At the same time, the genes specifically expressed in a cluster were also obtained. The hemocyte types of 11 clusters were identified by the highly expressed and/or specifically expressed genes. Based on the highly expressed genes in each hemocyte cluster, 11 clusters displayed cluster-specific highly expressed genes (**Figure 2A**), suggesting the different functions of hemocyte clusters at single-cell resolution.

SC0, the largest cluster of shrimp hemocytes (**Figure 1B**), was enriched in the genes encoding pre-rRNA, myosin regulatory light chain 2 and profilin (**Figure 2B**). In mammals, the accumulation of rRNAs is a signature of active macrophages (7). In *Drosophila*, myosin regulatory light chain and profilin play important roles in encapsulation of hemocytes (7). Thus, SC0 might be a macrophage-like cluster of shrimp hemocytes related to encapsulation (**Table 1**).

**Table 1:** Summary of shrimp hemocyte clusters.

Cluster	Signature gene	Hemocyte type
SC0	Pre-rRNA, myosin regulatory light chain 2, profilin	Macrophage-like hemocytes related to encapsulation.
SC1	Pre-rRNA, alpha2-macroglobulin	Macrophage-like hemocytes
SC2	Mannose-binding protein, kazal-type proteinase inhibitor, serine protease, prophenoloxidase activating factor	Granular hemocytes involved in prophenoloxidase system regulation
SC3	C-type lysozyme, anti- lipopolysaccharide factor A1, anti-lipopolysaccharide factor-like protein	Semi-granular hemocytes involved in antimicrobial peptide production
SC4	C-type lectin, β-integrin	Hyalinocytes
SC5	Prophenoloxidase, stylicin, penaeidin-II, mannose-binding protein	Granular hemocytes responsible for the production of antimicrobial peptides
SC6	Calreticulin, ribophorin I, serine proteinase inhibitor-3, C-type lectin	Intermediate hemocytes between semi-granular hemocytes and hyalinocytes related to cell proliferation
SC7	Pre-rRNA, lipoprotein receptor 1	Adipohemocyte-like hemocytes
SC8	Cortical rod-like protein, thrombospondin	Reproductive hemocytes
SC9	Hemocyte transglutaminase, single VWC domain protein 5, insulin-like growth factor-binding protein	Hyalinocyte hemocytes related to immune activation and cell differentiation
SC10	Hemocyte transglutaminase, proliferating cell nuclear antigen, cell division cycle 2	Hyalinocyte hemocytes related to cell proliferation

In SC1, alpha2-macroglobulin and the rRNA genes encoding pre-rRNA were highly expressed (**Figure 2B**). Alpha2-macroglobulin of shrimp is responsible for clotting pathway against pathogen infection (20). In mammals, the accumulation of rRNAs is a signature of active macrophages (7). In *Drosophila* hemocytes, plasmatocytes, similar to vertebrate macrophages, are enriched in rRNA genes (7). In this context, SC1 might be a macrophage-like cluster of shrimp hemocytes (**Table 1**).

The hemocyte cluster SC2 was enriched in the transcripts of mannose-binding protein, serine protease, kazal-type proteinase inhibitor and prophenoloxidase activating factor (**Figure 2B**). As reported, mannose-binding protein is the potential marker of granular hemocytes (21). Serine proteases are mainly expressed in granular cells (22). Kazal-type proteinase inhibitor and prophenoloxidase activating factor are important components in prophenoloxidase system of crustaceans (22). Thus SC2 might represent granular hemocytes involved in prophenoloxidase system regulation (**Table 1**).

In SC3, antimicrobial peptide genes, including C-type lysozyme and anti- lipopolysaccharide factor A1, and anti-lipopolysaccharide factor-like protein, were highly expressed (**Figure 2B**). It is reported that antimicrobial peptide genes are highly expressed in semi-granular cells (22). Therefore, SC3 could be semi-granular hemocytes, being responsible for the production of antimicrobial peptides (**Table 1**).

SC4 was enriched in the genes encoding C-type lectin and β-integrin (**Figure 2B**). As reported, integrins and C-type lectins were mainly expressed in hyalinocytes (22). In this context, SC4 represented a subcluster of hyalinocytes (**Table 1**).

SC5 was enriched in the genes encoding prophenoloxidase, stylicin, penaeidin-II and mannose-binding protein (**Figure 2B**). As reported, mannose-binding protein (mannose-binding lectin, MBL), the key protein for the mannose-binding lectin-mediated complement activation pathway, is exclusively expressed in granular cells (23). Prophenoloxidase is mainly expressed in granular cells (19), while stylicin and penaeidin-II are important antimicrobial peptides involved in crustacean humoral immunity (24). Therefore, SC5 might represent granular hemocytes responsible for the production of antimicrobial peptides (**Table 1**).

In SC6, the calreticulin, ribophorin I, C-type lectin and serine proteinase inhibitor-3 genes were highly expressed (**Figure 2B**). As reported, calreticulin plays important roles in shrimp antiviral immunity *via* MjsvCL (*M. japonicus* stomach virus–associated C-type lectin)–calreticulin pathway (25), as well as functions in cellular proliferation in cancer (26). Ribophorin I, a transmembrane glycoprotein, is associated with the cellular proliferation in vertebrates and antiviral immunity in shrimp (27). C-type lectins were mainly expressed in hyalinocytes, while most of serine proteinase inhibitors exhibited high expression levels in semi-granular cells (22). Thus SC6 represented intermediate hemocytes between semi-granular hemocytes and hyalinocytes related to cell proliferation (**Table 1**).

The hemocyte cluster SC7 was enriched in rRNA genes and lipoprotein receptor 1 gene (**Figure 2B**). In the activated macrophages, rRNA genes are highly expressed (7). Lipoprotein receptor, a lipid metabolism-related protein (28), is enriched in the adipohemocytes of insects (29). Adipohemocytes, enriched in lipid droplets, are comparable to the lipid-containing macrophages of vertebrates (29). Thus, SC7 might adipohemocyte-like hemocytes comparable to macrophages (**Table 1**).

In SC8, the genes encoding cortical rod-like protein and thrombospondin were highly expressed (**Figure 2B**). As reported, the cortical rod protein and thrombospondin are enriched in the ovary of shrimp *Marsupenaeus japonicus* (30). Therefore, SC8 might represent reproductive hemocytes (**Table 1**).

SC9 was enriched in the genes encoding hemocyte transglutaminase, single VWC (von Willebrand factor type C) domain protein 5 (Vago5) and insulin-like growth factor-binding protein (**Figure 2B**). Transglutaminase of crustacean, similar to mammalian factor XIIIa, is a marker of immature hemocytes and mainly expressed in hyalinocytes, which functions in regulating the blood coagulation and stabilizing the extracellular matrix proteins (20, 31, 32). Single VWC domain protein 5, an interferon-like molecule, plays an important role in activating the shrimp antiviral immunity (20). Insulin-like growth factor-binding protein is essential for cell differentiation and organ maturation in shrimp (20). Therefore, SC9 might represent hyalinocyte hemocytes related to immune activation and cell differentiation (**Table 1**).

In SC10, the genes encoding hemocyte transglutaminase, proliferating cell nuclear antigen and cell division cycle 2 were highly expressed (**Figure 2B**). The high expression level of hemocyte transglutaminase suggested that SC10 was hyalinocyte hemocytes. Proliferating cell nuclear antigen is a molecular marker for prawn hematopoietic stem cells, as well as the potential marker of hyalinocytes (33). Cell division cycle 2, also termed as cyclin-dependent kinase 1, is essential for cell proliferation and self-renewal of stem cells (34). In this context, SC10 might be hyalinocyte hemocytes related to cell proliferation (**Table 1**).

Collectively, these findings demonstrated that the shrimp hemocytes consisted of 11 types of hemocytes.

### The shrimp hemocytes responsible for immunity

**Figure 3:**
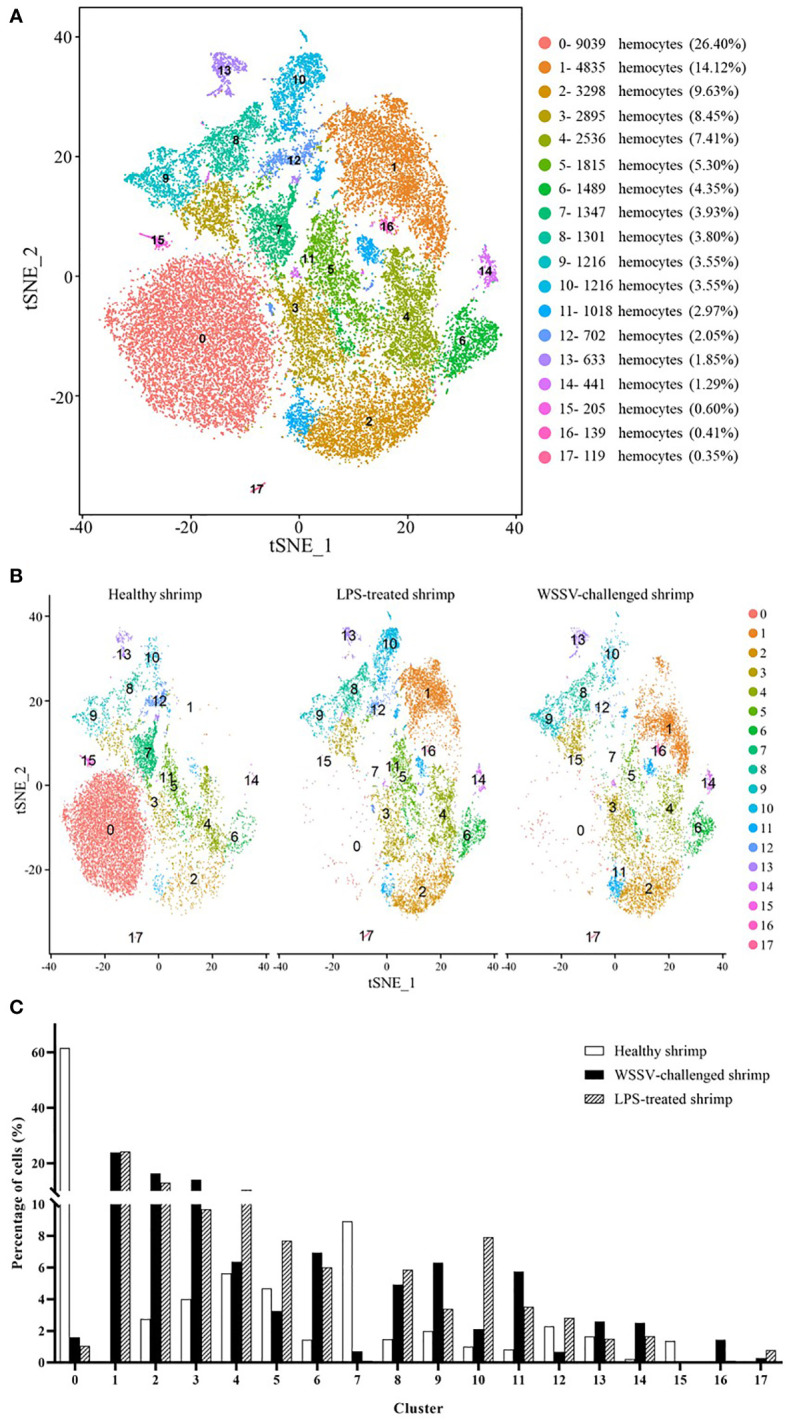
The shrimp hemocytes responsible for immunity. **(A)** The clusters of hemocytes from the combined single-cell RNA-seq data of healthy shrimp, WSSV-challenged shrimp and LPS-treated shrimp by t-SNE analysis. A total of 34,244 hemocytes were characterized. The different types of hemocytes were indicated with different colors. The axes corresponded to the 2-dimensional embedding produced by the t-SNE algorithm. **(B)** t-SNE displaying all identified hemocyte clusters in healthy shrimp, WSSV-challenged shrimp and LPS-treated shrimp. **(C)** Percentage of hemocyte clusters in healthy, WSSV-challenged and LPS-treated shrimp.

To further reveal the hemocytes responsible for shrimp immunity, shrimp were challenged by WSSV or LPS, followed by single-cell RNA sequencing (**Figure 1A**). Then three single cell RNA-seq data, including healthy shrimp, WSSV-challenged shrimp and LPS-treated shrimp, were merged to cluster the hemocytes presenting similar gene expression profiles. A total of 34,244 hemocytes (healthy shrimp, 14,258; WSSV-challenged shrimp, 8,816; LPS-treated shrimp, 11,170) were profiled into 18 clusters (**Figure 3A**) (named from SC0-merged to SC17-merged) (GenBank accession no. PRJNA913773).

To estimate cell fraction changes, three conditions were segregated and visualized with t-SNE (**Figure 3B**). Then the cell fractions of different conditions in each cluster were calculated. In the WSSV-challenged shrimp, the proportion of cells in 3 clusters (SC4-merged, SC5-merged and SC13-merged) did not change more than 2 folds compared to those in healthy shrimp (**Figure 3C**), indicating that these 3 hemocyte clusters were not involved in shrimp immunity against viral infection. In the LPS-treated shrimp, 5 clusters (SC4-merged, SC5-merged, SC9-merged, SC12-merged and SC13-merged) did not have 2 folds more or less cells compared to healthy shrimp (**Figure 3C**), showing that these 5 clusters might not function in shrimp immunity against bacterial infection. These data indicated that three clusters (SC4-merged, SC5-merged and SC13-merged) were not involved in shrimp immunity. Based on cell-free barcodes, the clusters SC2, SC4 and SC10 of healthy shrimp, respectively corresponding to SC5-merged, SC4-merged and SC13-merged, were not immune hemocytes in shrimp. A total of 15 clusters, including SC0-merged, SC1-merged, SC2-merged, SC3-merged, SC6-merged, SC7-merged, SC8-merged, SC9-merged, SC10-merged, SC11-merged, SC12-merged, SC14-merged, SC15-merged, SC16-merged and SC17-merged, were responsible for shrimp immunity, of which the clusters SC9-merged and SC12-merged were specific for shrimp immunity against to virus infection. Based on cell-specific barcodes (**Table 2**), therefore, SC0, SC1, SC3, SC5, SC6, SC7, SC8 and SC9 were the immune hemocytes of shrimp, of which SC6 was specific to virus infection. However, no cluster was specific to bacteral infection. Among the immune hemocytes of shrimp, the percentage of cells in SC0 in WSSV-challenged or LPS-treated shrimp was sharply decreased compared with that in healthy shrimp (**Figure 3C**), suggesting that SC0 might represent a prohemocyte cluster and SC0 might be differentiated into different hemocyte subsets of shrimp in response to pathogen infection.

**Table 2:** The immune hemocyte clusters of shrimp.

Clusters in merged set	Shrimp	Clusters in merged set	Shrimp
SC0-merged	SC0	SC10- merged	SC9
SC1- merged	/	SC11-merged	SC5
SC2- merged	SC5	SC12- merged	SC6
SC3-merged	SC3	SC14-merged	/
SC6- merged	SC5	SC15-merged	SC8
SC7-merged	SC1, SC7	SC16- merged	/
SC8- merged	SC3	SC17- merged	/
SC9-merged	SC3		

Taken together, these findings revealed that shrimp hemocytes SC0, SC1, SC3, SC5, SC6, SC7, SC8 and SC9 functioned in shrimp immunity against pathogen infection.

### Classification of the clusters of crayfish hemocytes by single-cell RNA sequencing

To further identify the hemocyte types of crustaceans, *Procambarus clarkii*, a kind of typical freshwater crayfish, was characterized by single-cell RNA sequencing (**Figure 1A**). Because of the lack of complete sequence of crayfish genome, the full-length transcriptome of crayfish hemocytes using single-molecule real-time (SMRT) sequencing was conducted. A total of 16,027 full-length mRNAs and 761 long non-coding RNAs (lncRNAs) were obtained (GenBank accession number PRJNA894120).

**Figure 4:**
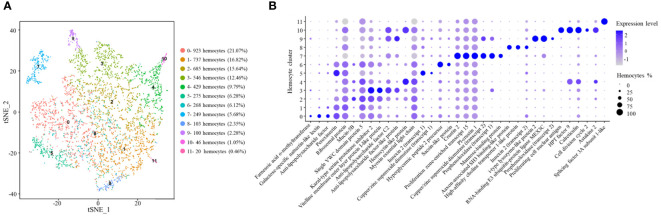
Classification of the clusters of crayfish hemocytes by single-cell RNA sequencing. **(A)** The clusters of hemocytes of healthy crayfish by t-SNE analysis. A total of 4,381 hemocytes were characterized. The different types of hemocytes were indicated with different colors. The axes corresponded to the 2-dimensional embedding produced by the t-SNE algorithm. **(B)** Dot plots showing the highly expressing genes in hemocyte clusters. Color gradient of the dot represented the expression level, and the dot size indicated the percentage of hemocytes expressing the genes.

Based on single-cell RNA-seq, the hemocytes of crayfish were classified into 12 clusters (**Figure 4A**) (named from CC0 to CC11) (GenBank accession no. PRJNA913786). CC0 was enriched in the genes encoding farnesoic acid O-methyltransferase, galactose-specific nattectin-like lectin, anti-lipopolysaccharide factor and peroxinectin (**Figure 4B**). As reported, farnesoic acid O-methyltransferase, a key enzyme involved in methyl farnesoate (a crustacean equivalent of insect juvenile hormone) synthesis, is upregulated after *Vibrio anguillarum* and WSSV challenge (35). Galactose-specific nattectin-like lectin is a kind of C-type lectin which functions in antibacterial defense (36). However, this protein has not been reported in crustaceans. Anti-lipopolysaccharide factor is mainly expressed in semi-granular cells in shrimp (22). Peroxinectin, a cell adhesion molecule responsible for degranulation, encapsulation and enhancement, exhibits the highest expression level in granular hemocytes (31). Thus, CC0 might represent an intermediate hemocytes between semi-granular and granular hemocytes.

CC1 had a similar gene expression profiles of anti-lipopolysaccharide factor and peroxinectin to those of CC0 (**Figure 4B**). Besides, CC1 highly expressed ribosomal protein. In shrimp, several ribosomal genes are significantly upregulated in stress response to support the synthesis of stress defense protein (37). The results suggested that CC2 might be an intermediate hemocytes between semi-granular and granular hemocytes associated with stress response.

CC2 was enriched in the transcripts of Kazal-type serine proteinase inhibitor 2, mucin-5B and single VWC (von Willebrand factor type C) domain protein 5 (**Figure 4B**). The protein kazal-type proteinase inhibitor 2 (hcPcSPI2) is a potential marker of semi-granular hemocytes in *Procambarus clarkii* and *Pacifastacus leniusculus* (38). Mucins are mainly involved in mucosal immune responses in intestine against pathogens (39), while single VWC domain protein 5 (Vago5) is related to the activation of antiviral immunity (20). In this context, CC2 might represent semi-granular hemocytes functioned in mucosal immunity.

In the hemocyte cluster CC3, the genes encoding vitelline membrane outer layer protein I-like protein, anti-lipopolysaccharide factor C2, kazal-type proteinase inhibitor 2 and anti-lipopolysaccharide factor-like protein were highly expressed (**Figure 4B**). Kazal-type proteinase inhibitor 2 is the marker of semi-granular hemocytes in crayfish (38). Vitelline membrane outer layer protein I, a potential marker of mature hemocytes, can be detected both in granular and semi-granular hemocytes in signal crayfish *Pacifastacus leniusculus* (40). Anti-lipopolysaccharide factor is the antimicrobial peptide mainly expressed in semi-granular hemocytes of shrimp (22). Therefore, CC3 might represent the mature semi-granular hemocytes responsible for the production of antimicrobial peptides.

CC4 was enriched in the genes encoding hemocytin-like protein and myosin essential light chain (**Figure 4B**). Hemocytin is an anti-bacterial protein involved in innate immune system of *Eriocheir sinensis* and *Bombyx mori* (41), while myosin light chain is requirement for hemocytic phagocytosis in shrimp (42). Thus CC4 might represent a hemocyte cluster related to phagocytosis.

In CC5, copper/zinc superoxide dismutase and innexin 2 genes were highly expressed (**Figure 4B**). In signal crayfish *Pacifastacus leniusculus*, copper/zinc superoxide dismutase is well known as the marker of granular hemocytes (19). However, other markers of granular hemocytes, such as mannose-binding protein are negligibly detected in CC5, indicating CC5 might be immature granular hemocytes. Innexin, a gap junction protein, functions as an antiviral protein in *Procambarus clarkia* (43). Therefore, CC5 might be immature granular hemocytes involved in immune responses.

CC6 was enriched in the genes encoding hyperglycemic hormone peptide 2 precursor and sacsin-like protein (**Figure 4B**). Hyperglycemic hormone peptide 2 precursor, mainly in granular cells and semi-granular cells (44), is a polypeptide hormone involved in environmental stress (36). In humans, sacsin functions as a regulator of heat shock protein 70 (Hsp70), a stress protein involved in environmental stress (45). In this context, CC6 might be an intermediate hemocytes between semi-granular and granular hemocytes associated to environmental stimulation.

CC7 was enriched in the transcripts of copper/zinc superoxide dismutase, prophenoloxidase, mannose-binding protein, PI-crustin 1, crustin 2 and proliferation zone-enriched transcript 15 (PET-15) (**Figure 4B**). As reported, copper/zinc superoxide dismutase and mannose-binding protein are the markers of granular hemocytes in crayfish *Pacifastacus leniusculus*, while prophenoloxidase is mainly expressed in granular hemocytes of crayfish (19). Crustins are antimicrobial peptides (24). PET-15 is a putative antimicrobial protein similar to crustin (46). Therefore, CC7 represented granular hemocytes responsible for the production of antimicrobial peptides.

In CC8, the genes encoding axon-associated SH3 binding-like protein, high-affinity choline transporter 1-like protein and innexin 2 were highly expressed (**Figure 4B**). In crayfish, axon-associated SH3 binding-like protein is expressed abundantly in neurons (47). High-affinity choline transporter 1 is involved in choline transport from the extracellular space to the neuron (48). Innexin 2 is required for the regulation of neuronal networks (49). Thus, CC8 might represent the hemocyte cluster related to nervous system.

CC9 was enriched in copper/zinc superoxide dismutase, prophenoloxidase, i-type lysozyme-like protein 2 and RNA-binding E3 ubiquitin-protein ligase MEX3C. Copper/zinc superoxide dismutase and prophenoloxidase are the markers of granular hemocytes and mature hemocytes of crustaceans, respectively (19, 20). I-type lysozymes are typical invertebrate antibacterial proteins involved in innate immunity of crustaceans (50). In humans, RNA-binding E3 ubiquitin-protein ligase MEX3C is highly expressed in activated NK cells (51). Therefore, CC9 might be granular hemocytes involved in immune responses.

CC10 exhibited high expression levels of proliferating cell nuclear antigen, cell division cycle 2, HPT factor 9, calreticulin and astakine 2 genes. As reported, proliferating cell nuclear antigen is the marker of hyalinocytes in *Pacifastacus leniusculus* (33). Cell division cycle 2 is essential for cell proliferation in mammalian cells (34). HPT factor 9 is a cytokine related to hematopoietic tissue. Astakines are hematopoietic cytokines in invertebrates and vertebrates (21). Calreticulin is important for cell proliferation (26). In this context, CC10 might represent hyalinocytes of crayfish.

CC11 was enriched in the gene encoding splicing factor 3A subunit 1-like protein (SF3A1) (**Figure 4B**). SF3A1 is a marker of natural killer T cells (52). Therefore, CC11 might be a natural killer T cell-like cluster.

Collectively, the signature genes and putative hemocyte types of crayfish were summarized in **Table 3**.

**Table 3:** Summary of crayfish hemocyte clusters.

Cluster	Signature gene	Hemocyte type
CC0	Farnesoic acid O-methyltransferase, galactose-specific nattectin-like lectin, anti-lipopolysaccharide factor, peroxinectin	Intermediate hemocytes between semi-granular and granular hemocytes
CC1	Anti-lipopolysaccharide factor, peroxinectin, ribosomal protein	Intermediate hemocytes between semi-granular and granular hemocytes
CC2	Kazal-type serine proteinase inhibitor 2, mucin-5B, single VWC domain protein 5	Semi-granular hemocytes functioned in mucosal immunity
CC3	Vitelline membrane outer layer protein I-like protein, anti- lipopolysaccharide factor C2, kazal-type proteinase inhibitor 2, anti-lipopolysaccharide factor-like protein	Mature semi-granular hemocytes responsible for the production of antimicrobial peptides
CC4	Hemocytin-like protein, myosin essential light chain	Hemocytes related to phagocytosis
CC5	Copper/zinc superoxide dismutase, innexin 2	Immature granular hemocytes involved in immune responses
CC6	Hyperglycemic hormone peptide 2 precursor, sacsin-like protein	Intermediate hemocytes between semi-granular and granular hemocytes associated to environmental stimulation
CC7	Copper/zinc superoxide dismutase, prophenoloxidase, mannose-binding protein, PI-crustin 1, crustin 2, proliferation zone-enriched transcript 15	Granular hemocytes involved in the production of antimicrobial peptides
CC8	Axon-associated SH3 binding-like protein, high-affinity choline transporter 1-like protein, innexin 2	Hemocytes related to nervous system
CC9	Copper/zinc superoxide dismutase, prophenoloxidase, i-type lysozyme-like protein 2, RNA-binding E3 ubiquitin-protein ligase MEX3C	Granular hemocytes involved in immune responses.
CC10	Proliferating cell nuclear antigen, cell division cycle 2, astakine 2, HPT factor 9, calreticulin	Hyalinocytes
CC11	Splicing factor 3A subunit 1-like protein	Natural killer T cell-like cluster

### The immunity-associated hemocytes of crayfish

**Figure 5:**
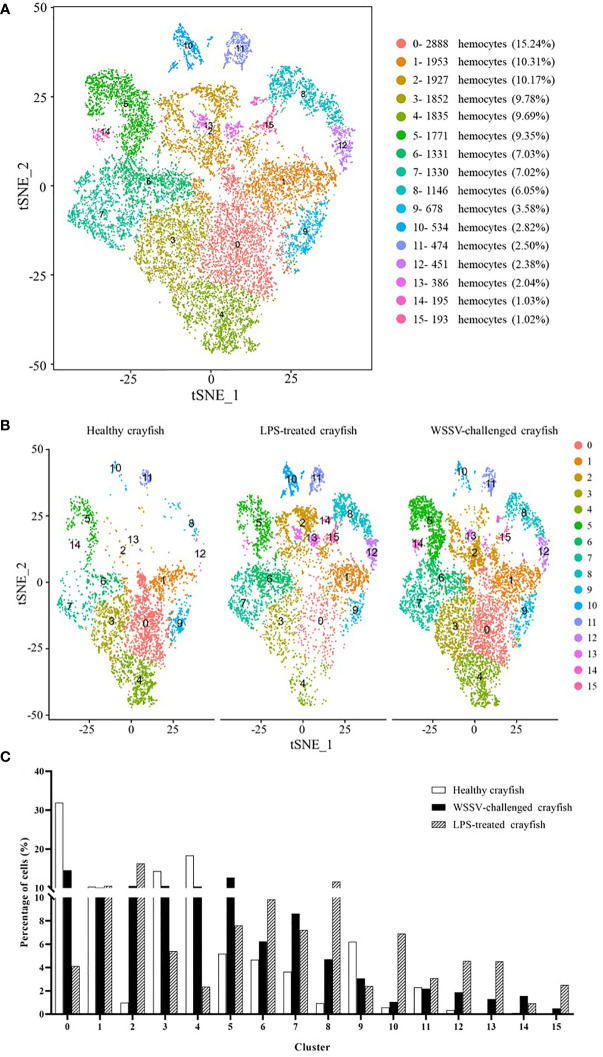
The immunity-associated hemocytes of crayfish. **(A)** The clusters of crayfish hemocytes using the merged single cell RNA-seq data of healthy crayfish, WSSV-challenged crayfish and LPS-treated crayfish by t-SNE analysis. A total of 18,944 hemocytes were characterized. The different types of hemocytes were indicated with different colors. The percentage of each cluster was shown in the parenthesis. The axes corresponded to the 2-dimensional embedding produced by the t-SNE algorithm. **(B)** t-SNE displaying all the identified hemocyte clusters in healthy, WSSV-challenged and LPS-treated crayfish. **(C)** Percentage of each cluster in the hemocytes of healthy, WSSV-challenged or LPS-treated crayfish.

To further identify the hemocytes involved in the immune responses of crayfish, the single cell RNA-seq data of healthy crayfish, WSSV-challenged crayfish and LPS-treated crayfish were merged (**Figure 1A**). The merged data generated 16 clusters of crayfish hemocytes (**Figure 5A**) (named from CC0-merged to CC15-merged) (GenBank accession no. PRJNA913786). Among the 16 clusters, clusters 0-11 were well detected in healthy crayfish, WSSV-challenged crayfish and LPS-treated crayfish, while clusters 12-15 mainly emerged in the hemocytes of WSSV or/and LPS-challenged crayfish, indicating that clusters 12-15 were the newly differentiated hemocyte populations in response to LPS and WSSV challenge (**Figure 5B**). Clusters 12-15 might be responsible for immunity of crayfish.

To further distinguish the immune and non-immune cell clusters, the percentages of the hemocyte fraction alterations in healthy crayfish, WSSV-challenged crayfish and LPS-treated crayfish were compared. It was found that the percentages of cells in clusters 2, 5, 7, 8, 12, 13, 14 and 15 in the WSSV-challenged or the LPS-treated crayfish were 2 folds more than those of the healthy crayfish (**Figure 5C**), showing that clusters 2, 5, 7, 8, 12, 13, 14 and 15 might be activated in crayfish immunity.

To reveal the relationship between the hemocyte clusters of healthy crayfish and the merged hemocyte clusters of crayfish, the hemocytes from healthy crayfish in the merged set were mapped to the clusters in the separate set of healthy crayfish using cell-specific barcodes. The results showed that the hemocytes of 2 clusters CC1-merged and CC11-merged, which did not show a striking depletion or increase upon WSSV infection or/and LPS stimulation (**Figure 5C**), mainly clustered in CC4 and CC8 of healthy crayfish, indicating that hemocyte CC4 and CC8 might be non-immune cell clusters in healthy crayfish. The hemocytes CC0-merged, CC2-merged, CC3-merged, CC4-merged, CC5-merged, CC6-merged, CC7-merged, CC8-merged, CC9-merged and CC10-merged mainly clustered in CC0, CC1, CC2, CC3, CC5, CC6, CC7, CC9, CC10 and CC11 of healthy crayfish (**Table 4**), suggesting that hemocytes CC0, CC1, CC2, CC3, CC5, CC6, CC7, CC9, CC10 and CC11 might be involved in the immune responses of crayfish, of which CC7 and CC9 were specific to viral infection and clusters CC5 and CC11 were specific to bacteral infection.

**Table 4:** The immune hemocyte clusters of crayfish.

Clusters in merged set	crayfish	Clusters in merged set	crayfish
CC0-merged	CC0, CC1, CC2	CC8- merged	CC10
CC2- merged	CC0	CC9-merged	CC6
CC3-merged	CC0, CC2, CC3	CC10- merged	CC11
CC4-merged	CC0, CC5	CC12- merged	/
CC5-merged	CC7	CC13-merged	/
CC6- merged	CC3	CC14-merged	/
CC7-merged	CC3, CC9	CC15-merged	/

### Identification of crustacean immune hemocytes

**Figure 6:**
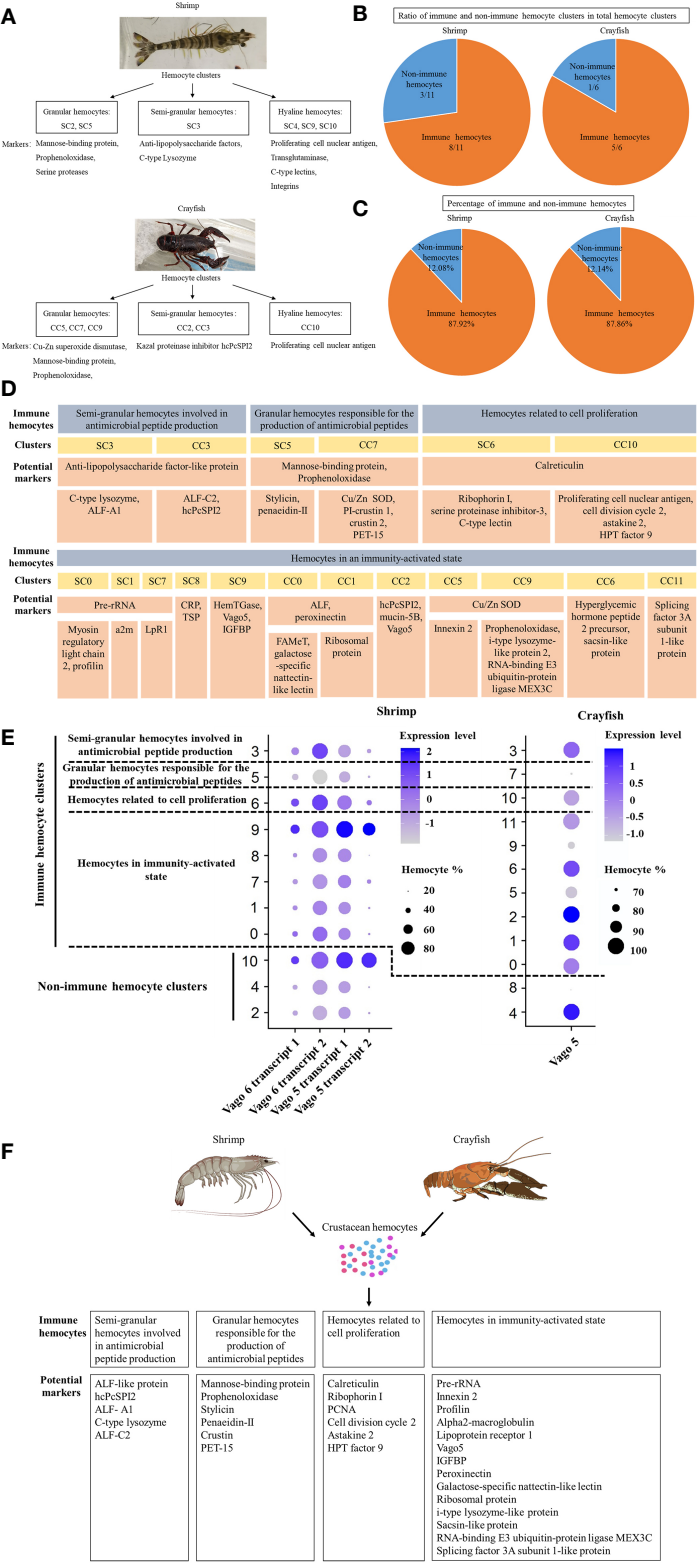
Identification of crustacean immune hemocytes. **(A)** The morphologically typical hemocyte subgroups of crustaceans. The typical hemocyte subgroups (granular hemocytes, semi-granular hemocytes and hyaline hemocytes) of shrimp and crayfish were determined based on the single-cell RNA sequencing data. The potential marker genes of three subgroups were indicated. **(B)** The ratio of the immune hemocyte clusters in the total clusters of crustaceans. The immune and non-immune hemocyte clusters were identified based on the single cell RNA-seq data of shrimp and crayfish. **(C)** The percentage of immune hemocytes in the shrimp and crayfish hemocytes. **(D)** Immune hemocytes in crustaceans. The immune hemocytes of shrimp and crayfish were assigned to 4 types based on the significant and specific gene expression in each cluster. ALF-like protein, anti-lipopolysaccharide factor-like protein; ALF-A1, anti-lipopolysaccharide factor A1; ALF-C2, anti-lipopolysaccharide factor C2; hcPcSPI2, SGC-specific kazal proteinase inhibitor, Cu/Zn SOD, Cu-Zn superoxide dismutase; PET-15, proliferation zone-enriched transcript 15; a2m, alpha2-macroglobulin; LpR1, lipoprotein receptor 1; ALF, anti-lipopolysaccharide factor; CRP, cortical rod-like protein; TSP, thrombospondin; HemTGase, hemocyte transglutaminase; Vago5, single VWC domain protein 5; FAMeT, farnesoic acid O-methyltransferase; PCNA, proliferating cell nuclear antigen; IGFBP, insulin-like growth factor-binding protein. **(E)** Dot plots profiling of genes of Vago family in each cluster of shrimp and crayfish. Color gradient of the dot represented the expression level, while the dot size indicated the percentage of hemocytes expressing the Vago genes. **(F)** Schematic diagram of immune hemocytes in crustacean.

To identify the morphologically typical hemocyte subgroups of crustaceans, including granular cells (GCs), semi-granular cells (SGCs) and hyaline cells (HCs), the hemocyte clusters of shrimp and crayfish were characterized. The results showed that the shrimp hemocyte clusters SC2 and SC5 belonged to granular hemocytes of shrimp, in which the markers (mannose-binding protein, prophenoloxidase and serine protease) of granular hemocytes were significantly upregulated (**Figure 6A**). The granular hemocytes of crayfish consisted of crayfish hemocyte clusters CC5, CC7 and CC9, highly expressing Cu-Zn superoxide dismutase, mannose-binding protein and prophenoloxidase, the markers of granular hemocytes (**Figure 6A**). The shrimp hemocyte cluster SC3 was semi-granular hemocytes of shrimp, while the crayfish hemocyte clusters CC2 and CC3 belonged to semi-granular hemocytes of crayfish, in which the markers of semi-granular hemocytes were upregulated (**Figure 6A**). The hyaline hemocytes of shrimp and crayfish consisted of SC4, SC9, SC10, and CC10, respectively (**Figure 6A**). The granular cells, semi-granular cells and hyaline cells accounted for 22.3% and 43.39% of the hemocytes of shrimp and crayfish, respectively.

To further identify the hemocytes involved in immune responses of crustacean, the immunity-related hemocyte clusters of shrimp and crayfish were analyzed. The results showed that the ratio of the immune hemocyte clusters in the total clusters of shrimp or crayfish accounted for 8/11 or 5/6 (**Figure 6B**). At the same time, the immune hemocytes of shrimp or crayfish accounted for 87.92% or 87.86% of the total number of hemocytes (**Figure 6C**). These data indicated that most of hemocyte clusters of crustaceans were associated with immunity. The immune hemocytes of crustaceans were assigned to 4 types, including semi-granular hemocytes involved in antimicrobial peptide production (potential markers, anti-lipopolysaccharide factors and SGC-specific kazal proteinase inhibitor), granular hemocytes responsible for the production of antimicrobial peptides (potential markers, mannose-binding protein, prophenoloxidase, Cu-Zn superoxide dismutase, penaeidin and crustin), hemocytes related to cell proliferation (potential markers, proliferating cell nuclear antigen, cell division cycle 2 and calreticulin) and hemocytes in immunity-activated state (potential markers, pre-rRNA, innexin, profilin alpha2-macroglobulin and Vago5) (**Figure 6D**). It was found that the Vago family, the analogue of interferon, was enriched in three types of immune hemocytes including hemocytes in immunity-activated state, semi-granular hemocytes involved in antimicrobial peptide production and hemocytes related to cell proliferation (**Figure 6E**), indicating that these immune hemocytes were responsible for virus invasion.

Taken together, these findings revealed that the immune hemocytes of crustacean consisted of 4 types of hemocytes (semi-granular hemocytes involved in antimicrobial peptide production, granular hemocytes responsible for the production of antimicrobial peptides, hemocytes related to cell proliferation and hemocytes in immunity-activated state) (**Figure 6F**).

## Discussion

In invertebrates, hemocytes, referred to myeloid-like immune cells, are essential for the innate immune responses (7, 21). In *Drosophila*, the hemocytes are classified into three major subsets including plasmatocytes, crystal cells and lamellocytes (7). The mosquito hemocytes are divided into prohemocytes, phagocytic granulocytes and oenocytoids (53). In lepidopteran insects, prohemocytes, plasmatocytes, granulocytes, spherulocytes and oenocytoids are the main components of hemocytes (16). In crustaceans, the hemocytes are traditionally classified into three major types including granular cells, semi-granular cells and hyaline cells (21). However, the functional hemocytes of invertebrates cannot be classified based on the low-resolution cell morphologies (7). In this study, the results of single-cell RNA sequencing of shrimp and crayfish revealed that the hemocytes of crustaceans consisted of three classic hemocytes (granular hemocytes, semi-granular hemocytes and hyaline hemocytes) and novel subpopulations (macrophage-like hemocytes, adipohemocyte-like hemocytes, reproductive hemocytes, hemocytes related to phagocytosis, hemocytes related to nervous system, and natural killer T cell-like cluster) and intermediate subpopulations (intermediate hemocytes between semi-granular hemocytes and hyalinocytes, and intermediate hemocytes between semi-granular and granular hemocytes). Recently the single-cell RNA sequencing analysis of shrimp was performed by other research groups (20, 37, 54, 55). Nevertheless three typical hemocytes (granular cells, semi-granular cells and hyaline cells) are not identified in the previous studies (20, 37, 54), showing the limitation of the previous investigations. The immunity-associated markers, including hemocyte transglutaminase, Vago 5, ALF-like, penaeidin-II, C-type lysozyme, stylicin, penaeidin-II, hemocyte kazal-type proteinase inhibitor (KPI), crustin, copper/zinc superoxide dismutase (SOD), prophenoloxidase and alpha2-macroglobulin, revealed in the present study, were also identified in the previous studies (20, 37, 54, 55). In this context, our study provided novel information on the classification of functional crustacean hemocytes.

Due to the lack of adaptive immunity in invertebrate, hemocytes are thought to be the most crucial cellular component of innate immunity for the recognition and elimination of foreign pathogens (56). Hemocytes not only participate in the cellular immunity including phagocytosis, melanization, clotting and coagulation, but also the humoral immunity *via* the production and release of different immune factors such as antimicrobial peptides, lectins and proteinase inhibitors (9, 21). Although the innate immunity of invertebrates depends on hemocytes, the immune cells of invertebrates remain unclassified at present. In this study, the hemocyte characterization at single-cell resolution revealed that the immune cells of crustaceans consisted of 4 types of hemocytes including semi-granular hemocytes involved in antimicrobial peptide production, granular hemocytes responsible for the production of antimicrobial peptides, hemocytes related to cell proliferation and hemocytes in immunity-activated state. Among these 4 types of immune hemocytes, 3 immune hemocytes (hemocytes related to cell proliferation, granular hemocytes involved in the production of antimicrobial peptides and hemocytes in immunity-activated state) contained the subpopulations that were specific to virus infection. In this context, our findings classified the immune cells of crustaceans for the first time, providing the solid basis for exploring crustacean immunity. The results of this investigation demonstrated that the hemocytes clusters had less significant changes in crayfish than in shrimp after the WSSV challenge (**Figures 3C**, **5C**). These data suggested that crayfish were more tolerant to WSSV than shrimp. The WSSV infection of shrimp could sharply alter the types of shrimp hemocytes. Although there existed 4 types of immune hemocytes in crustacean as revealed in this investigation, the biomarkers of these immune hemocytes remained to be further characterized in the future.

## Data availability statement

The data presented in the study are deposited in the GenBank repository, accession number PRJNA894118, PRJNA894120, PRJNA913773 and PRJNA913786.

## Author contributions

FX conducted experiments, analyzed data, and was a contributor in Figure creation and writing and preparation of the manuscript. XZ experimental design and concept, corresponding author, contributed in writing of the manuscript, major contributor in revision and preparation of the manuscript. All authors contributed to the article and approved the submitted version.

## References

[1] Pham LN Dionne MS Shirasu-Hiza M Schneider DS. A specific primed immune response in *Drosophila* is dependent on phagocytes. PLos Pathog (2007) 3:e26. doi: 10.1371/journal.ppat.0030026 17352533 PMC1817657

[2] Tormey CA Hendrickson JE. Transfusion-related red blood cell alloantibodies: Induction and consequences. Blood (2019) 133:1821–30. doi: 10.1182/blood-2018-08-833962 PMC6484385 30808636

[3] Rongey C Cherian S. Effects of chloroquine therapy on white blood cells. Blood (2015) 126:149. doi: 10.1182/blood-2015-05-642587 26539588

[4] Koupenova M Clancy L Corkrey HA Freedman JE. Circulating platelets as mediators of immunity, inflammation, and thrombosis. Circ Res (2018) 122:337–51. doi: 10.1161/CIRCRESAHA.117.310795 PMC5777300 29348254

[5] Vlisidou I Wood W. *Drosophila* blood cells and their role in immune responses. FEBS J (2015) 282:1368–82. doi: 10.1111/febs.13235 25688716

[6] Browne N Heelan M Kavanagh K. An analysis of the structural and functional similarities of insect hemocytes and mammalian phagocytes. Virulence (2013) 4:597–603. doi: 10.4161/viru.25906 23921374 PMC3906293

[7] Tattikota SG Cho B Liu Y Hu Y Barrera V Steinbaugh MJ. A single-cell survey of *Drosophila* blood. eLife (2020) 9:e54818. doi: 10.7554/eLife.54818 32396065 PMC7237219

[8] Feng M Swevers L Sun J. Hemocyte clusters defined by scRNA-seq in *Bombyx mori*: In silico analysis of predicted marker genes and implications for potential functional roles. Front Immunol (2022) 13:852702. doi: 10.3389/fimmu.2022.852702 35281044 PMC8914287

[9] Severo MS Landry JJM Lindquist RL Goosmann C Brinkmann V Collier P. Unbiased classification of mosquito blood cells by single-cell genomics and high-content imaging. Proc Natl Acad Sci U.S.A. (2018) 115:E7568–77. doi: 10.1073/pnas.1803062115 PMC6094101 30038005

[10] Wang W Zhang X. Comparison of antiviral efficiency of immune responses in shrimp. Fish Shellfish Immunol (2008) 25:522–7. doi: 10.1016/j.fsi.2008.07.016 18721886

[11] Fallon JP Reeves EP Kavanagh K. The *Aspergillus fumigatus* toxin fumagillin suppresses the immune response of *Galleria mellonella* larvae by inhibiting the action of haemocytes. Microbiol (Reading) (2011) 157:1481–8. doi: 10.1099/mic.0.043786-0 21349977

[12] Liu S Zheng SC Li YL Li J Liu HP. Hemocyte-mediated phagocytosis in crustaceans. Front Immunol (2020) 11:268. doi: 10.3389/fimmu.2020.00268 32194551 PMC7062681

[13] Defaye A Evans I Crozatier M Wood W Lemaitre B Leulier F. Genetic ablation of *Drosophila* phagocytes reveals their contribution to both development and resistance to bacterial infection. J Innate Immun (2009) 1:322–34. doi: 10.1159/000210264 20375589

[14] Van der Maaten LJP. Accelerating t-SNE using tree-based algorithms. J Mach Learn Res (2014) 15:3221–45.

[15] Stuart T Butler A Hoffman P Hafemeister C Papalexi E Mauck WM III. Comprehensive integration of single-cell data. Cell (2019) 177:1888–902. doi: 10.1016/j.cell.2019.05.031 PMC6687398 31178118

[16] Feng M Xia J Fei S Peng R Wang X Zhou Y. Identification of silkworm hemocyte subsets and analysis of their response to baculovirus infection based on single-cell RNA sequencing. Front Immunol (2021) 12:645359. doi: 10.3389/fimmu.2021.645359 33995363 PMC8119652

[17] Leitão AB Arunkumar R Day JP Geldman EM Morin-Poulard I Crozatier M. Constitutive activation of cellular immunity underlies the evolution of resistance to infection in. Drosophila eLife (2020) 9:e59095. doi: 10.7554/eLife.59095 33357377 PMC7785293

[18] Zhang X Yuan J Sun Y Li S Gao Y Yu Y. Penaeid shrimp genome provides insights into benthic adaptation and frequent molting. Nat Commun (2019) 10:356. doi: 10.1038/s41467-018-08197-4 30664654 PMC6341167

[19] Söderhäll I. Crustacean hematopoiesis. Dev Comp Immunol (2016) 58:129–41. doi: 10.1016/j.dci.2015.12.009 26721583

[20] Koiwai K Koyama T Tsuda S Toyoda A Kikuchi K Suzuki H. Single-cell RNA-seq analysis reveals penaeid shrimp hemocyte subpopulations and cell differentiation process. eLife (2021) 10:e66954. doi: 10.7554/eLife.66954 34132195 PMC8266392

[21] Lin X Söderhäll I. Crustacean hematopoiesis and the astakine cytokines. Blood (2011) 117:6417–24. doi: 10.1182/blood-2010-11-320614 21444913

[22] Sun M Li S Zhang X Xiang J Li F. Isolation and transcriptome analysis of three subpopulations of shrimp hemocytes reveals the underlying mechanism of their immune functions. Dev Comp Immunol (2020) 108:103689. doi: 10.1016/j.dci.2020.103689 32224106

[23] Wu C Charoensapsri W Nakamura S Tassanakajon A Söderhäll I Söderhäll K. An MBL-like protein may interfere with the activation of the proPO-system, an important innate immune reaction in invertebrates. Immunobiology (2013) 218:159–68. doi: 10.1016/j.devcel.2017.01.001 22459272

[24] Liu HT Wang J Mao Y Liu M Niu SF Qiao Y. Identification and expression analysis of a novel stylicin antimicrobial peptide from kuruma shrimp (*Marsupenaeus japonicus*). Fish Shellfish Immunol (2015) 47:817–23. doi: 10.1016/j.fsi.2015.09.044 26439413

[25] Wang XW Xu YH Xu JD Zhao XF Wang JX. Collaboration between a soluble c-type lectin and calreticulin facilitates white spot syndrome virus infection in shrimp. J Immunol (2014) 193:2106–17. doi: 10.4049/jimmunol.1400552 25070855

[26] Sun J Mu H Dai K Yi L. Calreticulin: a potential anti-cancer therapeutic target. Pharmazie (2017) 72:503–10. doi: 10.1691/ph.2017.7031 29441976

[27] Chotwiwatthanakun C Ngopon J Unajak S Jitrapakdee S. The ribophorin I from *Penaeus monodon* shrimp: cDNA cloning, expression and phylogenetic analysis. Comp Biochem Physiol B Biochem Mol Biol (2008) 150:331–7. doi: 10.1016/j.cbpb.2008.04.001 18479955

[28] Lee JH Kim BK Seo YI Choi JH Kang SW Kang CK. Four cDNAs encoding lipoprotein receptors from shrimp (*Pandalopsis japonica*): structural characterization and expression analysis during maturation. Comp Biochem Physiol B Biochem Mol Biol (2014) 169:51–62. doi: 10.1016/j.cbpb.2013.12.005 24389120

[29] Cho B Yoon SH Lee D Koranteng F Tattikota SG Cha N. Single-cell transcriptome maps of myeloid blood cell lineages in. Drosophila Nat Commun (2020) 11:4483. doi: 10.1038/s41467-020-18135-y 32900993 PMC7479620

[30] Kim YK Kawazoe I Jasmani S Ohira T Wilder MN Kaneko T. Molecular cloning and characterization of cortical rod protein in the giant freshwater prawn *Macrobrachium rosenbergii*, a species not forming cortical rod structures in the oocytes. Comp Biochem Physiol B Biochem Mol Biol (2007) 148:184–91. doi: 10.1016/j.cbpb.2007.05.008 17601759

[31] Yang CC Lu CL Chen S Liao WL Chen SN. Immune gene expression for diverse haemocytes derived from pacific white shrimp. Litopenaeus vannamei. Fish Shellfish Immunol (2015) 44:265–71. doi: 10.1016/j.fsi.2015.02.001 25681751

[32] Junkunlo K Söderhäll K Söderhäll I. Transglutaminase 1 and 2 are localized in different blood cells in the freshwater crayfish. Pacifastacus leniusculus Fish Shellfish Immunol (2020) 104:83–91. doi: 10.1016/j.fsi.2020.05.062 32479868

[33] Bouallegui Y. A comprehensive review on crustaceans' immune system with a focus on freshwater crayfish in relation to crayfish plague disease. Front Immunol (2021) 12:667787. doi: 10.3389/fimmu.2021.667787 34054837 PMC8155518

[34] Wang XQ Lo CM Chen L Ngan ES Xu A Poon RY. CDK1-PDK1-PI3K/Akt signaling pathway regulates embryonic and induced pluripotency. Cell Death Differ (2017) 24:38–48. doi: 10.1038/cdd.2016.84 27636107 PMC5260505

[35] Duan Y Liu P Li J Wang Y Li J Chen P. A farnesoic acid O-methyltransferase (FAMeT) from *Exopalaemon carinicauda* is responsive to *Vibrio anguillarum* and WSSV challenge. Cell Stress Chaperones (2014) 19:367–77. doi: 10.1007/s12192-013-0464-5 PMC3982035 24136172

[36] Zhang XW Yang CH Zhang HQ Pan XT Jin ZY Zhang HW. A c-type lectin with antibacterial activity in weather loach. Misgurnus anguillicaudatus J Fish Dis (2020) 43:1531–9. doi: 10.1111/jfd.13255 32924173

[37] Li Y Zhou F Yang Q Jiang S Huang J Yang L. Single-cell sequencing reveals types of hepatopancreatic cells and haemocytes in black tiger shrimp (*Penaeus monodon*) and their molecular responses to ammonia stress. Front Immunol (2022) 13:883043. doi: 10.3389/fimmu.2022.883043 35603188 PMC9114817

[38] Li XC Zhang RR Sun RR Lan JF Zhao XF Wang JX. Three kazal-type serine proteinase inhibitors from the red swamp crayfish *Procambarus clarkii* and the characterization, function analysis of hcPcSPI2. Fish Shellfish Immunol (2010) 28:942–51. doi: 10.1016/j.fsi.2010.02.011 20170735

[39] Duan Y Liu Q Wang Y Zhang J Xiong D. Impairment of the intestine barrier function in *Litopenaeus vannamei* exposed to ammonia and nitrite stress. Fish Shellfish Immunol (2018) 78:279–88. doi: 10.1016/j.fsi.2018.04.050 29709590

[40] Liu H Jiravanichpaisal P Söderhäll I Cerenius L Söderhäll K. Antilipopolysaccharide factor interferes with white spot syndrome virus replication *in vitro* and *in vivo* in the crayfish *Pacifastacus leniusculus* . J Virol (2006) 80:10365–71. doi: 10.1128/JVI.01101-06 PMC1641759 17041217

[41] Chen X Wang J Yue W Liu J Wang C. Hepatopancreas transcriptome analysis of Chinese mitten crab (*Eriocheir sinensis*) with white hepatopancreas syndrome. Fish Shellfish Immunol (2017) 70:302–7. doi: 10.1016/j.fsi.2017.08.031 28860074

[42] Liu W Han F Zhang X. Ran GTPase regulates hemocytic phagocytosis of shrimp by interaction with myosin. J Proteome Res (2009) 8:1198–206. doi: 10.1021/pr800840x 19166347

[43] Bao W Tang K Alcivar-Warren A. The complete genome of an endogenous nimavirus (*Nimav-1_LVa*) from the pacific whiteleg shrimp. Penaeus (Litopenaeus) Vannamei. Genes (2020) 11:94. doi: 10.3390/genes11010094 31947590 PMC7016691

[44] Wu SH Chen YJ Huang SY Tsai WS Wu HJ Hsu TT. Demonstration of expression of a neuropeptide-encoding gene in crustacean hemocytes. Comp Biochem Physiol A Mol Integr Physiol (2012) 161:463–8. doi: 10.1016/j.cbpa.2012.01.007 22269107

[45] Guo K Ruan G Fan W Wang Q Fang L Luo J. Immune response to acute heat stress in the intestine of the red swamp crayfish. Procambarus clarkii Fish Shellfish Immunol (2020) 100:146–51. doi: 10.1016/j.fsi.2020.03.017 32165247

[46] Stoss TD Nickell MD Hardin D Derby CD McClintock TS. Inducible transcript expressed by reactive epithelial cells at sites of olfactory sensory neuron proliferation. J Neurobiol (2004) 58:355–68. doi: 10.1002/neu.10294 14750148

[47] Dearborn RE Jr Szaro BG Lnenicka GA. Cloning and characterization of AASPs: novel axon-associated SH3 binding-like proteins. JJ Neurobio (1999) 38:581–94. doi: 10.1002/(sici)1097-4695(199903)38:4<581::aid-neu12>3.0.co;2-0 10084691

[48] Haga T. Molecular properties of the high-affinity choline transporter CHT1. J Biochem (2014) 156:181–94. doi: 10.1093/jb/mvu047 25073461

[49] Ducret E Alexopoulos H Le Feuvre Y Davies JA Meyrand P Bacon JP. Innexins in the lobster stomatogastric nervous system: Cloning, phylogenetic analysis, developmental changes and expression within adult identified dye and electrically coupled neurons. Eur J Neurosci (2006) 24:3119–33. doi: 10.1111/j.1460-9568.2006.05209.x 17156373

[50] Chen T Ren C Wang Y Luo P Jiang X Huang W. Molecular cloning, inducible expression and antibacterial analysis of a novel i-type lysozyme (lyz-i2) in pacific white shrimp. Litopenaeus vannamei. Fish Shellfish Immunol (2016) 54:197–203. doi: 10.1016/j.fsi.2016.04.008 27074443

[51] Cano F Bye H Duncan LM Buchet-Poyau K Billaud M Wills MR. The RNA-binding E3 ubiquitin ligase MEX-3C links ubiquitination with MHC-I mRNA degradation. EMBO J (2012) 31:3596–606. doi: 10.1038/emboj.2012.218 PMC3433784 22863774

[52] Young MD Mitchell TJ Vieira Braga FA Tran MGB Stewart BJ Ferdinand JR. Single-cell transcriptomes from human kidneys reveal the cellular identity of renal tumors. Science (2018) 361:594–9. doi: 10.1126/science.aat1699 PMC6104812 30093597

[53] Raddi G Barletta ABF Efremova M Ramirez JL Cantera R Teichmann SA. Mosquito cellular immunity at single-cell resolution. Science (2020) 369:1128–32. doi: 10.1126/science.abc0322 PMC8405044 32855340

[54] Cui C Tang X Xing J Sheng X Chi H Zhan W. Single-cell RNA-seq uncovered hemocyte functional subtypes and their differentiational characteristics and connectivity with morphological subpopulations in *Litopenaeus vannamei* . Front Immunol (2022) 13:980021. doi: 10.3389/fimmu.2022.980021 36177045 PMC9513592

[55] Yang P Chen Y Huang Z Xia H Cheng L Wu H. Single-cell RNA sequencing analysis of shrimp immune cells identifies macrophage-like phagocytes. eLife (2022) 11:e80127. doi: 10.7554/eLife.80127 36200862 PMC9584607

[56] Lv Z Qiu L Wang W Liu Z Liu Q Wang L. RGD-labeled hemocytes with high migration activity display a potential immunomodulatory role in the pacific oyster. Crassostrea gigas Front Immunol (2022) 13:914899. doi: 10.3389/fimmu.2022.914899 35865522 PMC9294365

