# Supplementary material for: Hallmarks of crustacean immune hemocytes at single-cell resolution
Source: Front Immunol. 2023 Jan 24;14:1121528. doi: 10.3389/fimmu.2023.1121528 (PMC9902875; doi:10.3389/fimmu.2023.1121528)
Supplement: Supplementary file 1 [file DataSheet_1.docx]

**SUPPLEMENTARY MATERIAL**

**FIGURE S1 A rich set of full-length cDNA sequences of *Marsupenaeus japonicus* revealed by single-molecule long-read sequencing.** (A) The annotation of the transcriptome in seven databases including NR (non-redundant), SwissProt, KEGG (Kyoto encyclopedia of genes and genomes), KOG (clusters of orthologous groups for eukaryotic complete genomes), GO (gene ontology), NT (nucleotide sequence database) and Pfam (Pfam protein domain database). (B) Species annotation in NR database. (C) GO classification of the putative functions of *Marsupenaeus japonicus* genes. (D) KEGG annotation of the putative functions of *Marsupenaeus japonicus* genes.


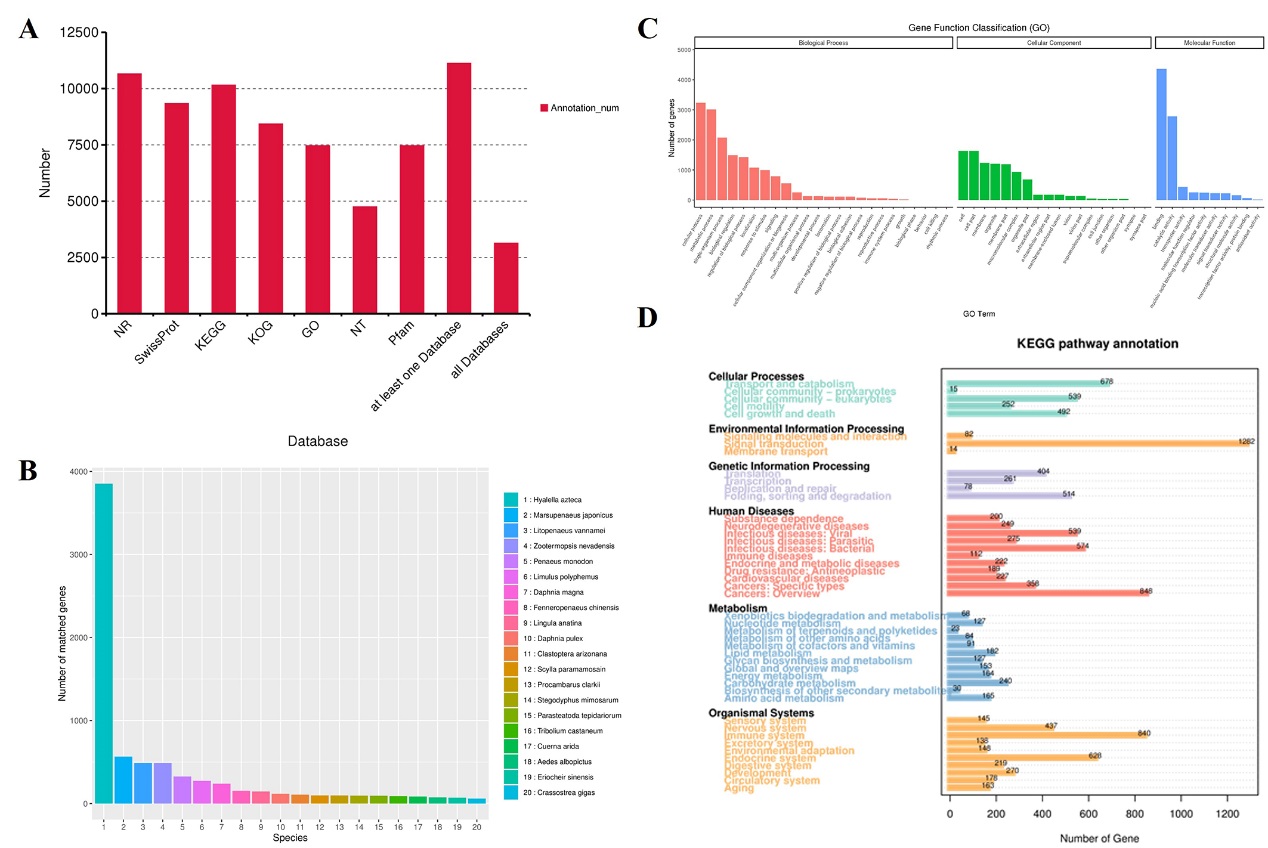


**TABLE S1. Reads of single-molecule real-time (SMRT) sequencing in *Marsupenaeus japonicus*.**

|  | Raw reads | Subreads | CSS reads | FLNC reads | Consensus reads | Genes by CD-HIT |
| --- | --- | --- | --- | --- | --- | --- |
| Number | 722,493 | 18,680,499 | 592,794 | 408,293 | 20,332 | 13,007 |
| Mean length (bp) | 59,594 | 2,227 | 3,169 | 2,987 | 3,220 | 3,314 |
